# Cyanobacteria from Terrestrial and Marine Sources Contain Apoptogens Able to Overcome Chemoresistance in Acute Myeloid Leukemia Cells

**DOI:** 10.3390/md12042036

**Published:** 2014-04-03

**Authors:** Liwei Liu, Lars Herfindal, Jouni Jokela, Tania Keiko Shishido, Matti Wahlsten, Stein Ove Døskeland, Kaarina Sivonen

**Affiliations:** 1 Division of Microbiology and Biotechnology, Department of Food and Environmental Sciences, University of Helsinki, P.O. Box 56, 00014 Helsinki, Finland; E-Mails: liwei.liu@helsinki.fi (L.L.); jouni.jokela@helsinki.fi (J.J.); tania.shishido@helsinki.fi (T.K.S.); matti.wahlsten@helsinki.fi (M.W.); 2 Department of Biomedicine, University of Bergen, Jonas Lies vei 91, N-5009 Bergen, Norway; E-Mails: lars.herfindal@biomed.uib.no (L.H.); stein.doskeland@biomed.uib.no (S.O.D.); 3 Translational Signaling Group, Haukeland University Hospital, Jonas Lies vei 91, N-5009 Bergen, Norway

**Keywords:** cyanobacteria, acute myeloid leukemia, hepatocyte, apoptosis, p53, Bcl-2, microcystin, nodularin

## Abstract

In this study, we investigated forty cyanobacterial isolates from biofilms, gastropods, brackish water and symbiotic lichen habitats. Their aqueous and organic extracts were used to screen for apoptosis-inducing activity against acute myeloid leukemia cells. A total of 28 extracts showed cytotoxicity against rat acute myeloid leukemia (IPC-81) cells. The design of the screen made it possible to eliminate known toxins, such as microcystins and nodularin, or known metabolites with anti-leukemic activity, such as adenosine and its analogs. A cytotoxicity test on human embryonic kidney (HEK293T) fibroblasts indicated that 21 of the 28 extracts containing anti-acute myeloid leukemia (AML) activity showed selectivity in favor of leukemia cells. Extracts L26-O and L30-O were able to partly overcome the chemotherapy resistance induced by the oncogenic protein Bcl-2, whereas extract L1-O overcame protection from the deletion of the tumor suppressor protein p53. In conclusion, cyanobacteria are a prolific resource for anti-leukemia compounds that have potential for pharmaceutical applications. Based on the variety of cellular responses, we also conclude that the different anti-leukemic compounds in the cyanobacterial extracts target different elements of the death machinery of mammalian cells.

## 1. Introduction

Acute myeloid leukemia (AML) is characterized by excess growth of leukemia cells and loss of normal blood cells. Leukemia cells have no infection-fighting capacity, thus AML patients become susceptible to infections, as well as bruising, bleeding and shortness of breath. Nowadays, the most common treatment regime for AML involves high doses of the cell cycle-specific inhibitor, cytarabine (Ara-C), in combination with the cell cycle unspecific inhibitor, anthracycline daunorubicin (DNR) [1]. Recently, a few promising drug leads have been discovered to fulfill the continuous need of new lead compounds. An inhibitor of the Fms-like tyrosine kinase3 (FLT3) showed promising activity in treatment trials of patients with acute myeloid leukemia [2]. Clofarabine, a nucleoside antimetabolite, was tested in Phase II clinical trial of pediatric patients with relapsed or refractory acute leukemia [3]. Gemtuzumab ozogamicin is a drug-linked monoclonal antibody, which is used to treat acute myelogenous leukemia. It was recently tested in a Phase II trial of therapy in older patients with acute myeloid leukemia [4]. However, chemoresistance usually limits AML therapy [5,6]. Thus, there is a need for novel anti-leukemia drugs, particularly to be able to treat patients who tolerate heavy chemotherapy poorly or who are found unsuited for stem cell transplantation.

Cyanobacteria are a large group of photosynthetic prokaryotic microorganisms, which commonly occur in a wide variety of environments, ranging from terrestrial, fresh, brackish to marine water. Some cyanobacteria live in symbioses with plants or fungi [7], and aquatic cyanobacteria are well known for forming blooms in eutrophic lakes and other aquatic environments. Many bloom-forming cyanobacteria produce toxins, such as microcystins and nodularin, which pose a health hazard to human and livestock [8,9,10,11]. However, cyanobacteria are able to produce a number of bioactive secondary metabolites having anti-tumor, anti-viral and anti-bacterial activities [12,13,14,15]. Due to the high diversity and chemical stability of many cyanobacterial natural products, they are promising candidates for pharmaceutical applications. Up to now, more than 40 anti-cancer biomolecules have been characterized from cyanobacteria [13], such as cryptophycins and curacins. Cryptophycins were tested in a Phase II trial for therapy against platinum ovarian cancer [16], and curacin A, which hinders tubulin polymerization [17], has been tested against breast cancer. There are many other compounds showing anti-cancer activity and potential for future testing in the clinical trials, such as dolastatin 10, malyngamide 3 and cocosamide B [18,19]. Marine cyanobacteria are considered a good source for novel bioactivities, and many potent bioactive compounds and drug leads have been isolated and identified, such as apratoxin A, bisebromoamide, coibamide and largazole [20,21,22,23]. In this study, our aim was to introduce an effective way to explore new anti-leukemia compounds of cyanobacteria isolated from biofilm, symbiotic lichen, mollusk association and brackish water habitats in order to find new drug leads for AML therapy.

## 2. Results and Discussion

### 2.1. Results

#### 2.1.1. Screening Cyanobacterial Extracts for Apoptosis-Inducing Activity

The cell line IPC-81, isolated from brown Norwegian acute myelocytic leukemia (BNML) rats, is a good model for AML *in vivo* and *in vitro* [24,25]. We therefore used these cells for the initial screen for apoptogenic activity from forty cyanobacteria strains. Eighteen strains were isolated and purified from biofilms from a rocky coastline, six from gastropods, two from a water plant, one from brackish water from the coastline of the Gulf of Finland and 13 from lichens (Table 1).

**Table 1:** The cyanobacteria strain studied. All *Nostoc* strains are lichen symbionts. Coordinates: 59°49ʹ55″ N, 23°5ʹ10″ E (Kobben) and 59°49ʹ11–22″ N, 22°58ʹ34″–59ʹ10″ E (Hanko Casino sea shore (HC)).

Code	Genus	Strain	Live habitat; Location
L1	*Calothrix*	HAN 24/1	Rock pond water, Kobben, Hanko, Finland
L2	*Calothrix*	HAN 33/2	Brown/yellow biofilm, HC, Hanko, Finland
L3	*Calothrix*	HAN 38/3	Red biofilm, HC, Hanko, Finland
L4	*Calothrix*	HAN 6/4	Growth on rock waterline, Kobben, Hanko, Finland
L5	*Calothrix*	HAN 17/1	Red biofilm, Kobben, Hanko, Finland
L6	*Calothrix*	HAN 21/4	Gastropod 10 cm under water, Kobben, Hanko, Finland
L7	*Anabaena*	HAN 15/1	Gastropod from waterline, Kobben, Hanko, Finland
L8	*Calothrix*	HAN 21/3	Gastropod 10 cm under water, Kobben, Hanko, Finland
L9	*Calothrix*	HAN 37/3	Green biofilm, HC, Hanko, Finland
L10	*Calothrix*	HAN 21/5	Gastropod 10 cm under water, Kobben, Hanko, Finland
L11	*Calothrix*	HAN 36/2	Biofilm, HC, Hanko, Finland
L12	*Calothrix*	HAN 22/1	Black biofilm, Kobben, Hanko, Finland
L13	*Calothrix*	HAN 22/2	Black biofilm, Kobben, Hanko, Finland
L14	*Calothrix*	HAN 6/3	Growth on rock waterline, Kobben, Hanko, Finland
L15	*Calothrix*	HAN 8/1	Biofilm, Kobben, Hanko, Finland
L16	*Calothrix*	HAN 38/2	Red biofilm, HC, Hanko, Finland
L17	*Calothrix*	HAN 3/19	Green biofilm, Kobben, Hanko, Finland
L18	*Calothrix*	HAN 33/1	Brown/yellow biofilm, HC, Hanko, Finland
L19	*Nodularia*	HAN 37/1	Green biofilm, HC, Hanko, Finland
L20	*Calothrix*	HAN 16/1	Waterplant from shallow water, Kobben, Hanko, Finland
L21	*Calothrix*	HAN 37/2	Green biofilm, HC, Hanko, Finland
L22	*Calothrix*	HAN 26/2	Black biofilm, HC, Hanko, Finland
L23	*Calothrix*	HAN 30/2	Green biofilm and Gastropod, HC, Hanko, Finland
L24	*Calothrix*	HAN 20/2	Black grains from rock surface, Kobben, Hanko, Finland
L25	*Anabaena*	HAN 15/2	Gastropod from waterline, Kobben, Hanko, Finland
L26	*Anabaena*	HAN 21/1	Gastropod 10 cm under water, Kobben, Hanko, Finland
L27	*Calothrix*	HAN 16/2	Waterplant from shallow water, Kobben, Hanko, Finland
L28	*Nostoc*	UK 2aImII	*Peltigera* sp. Helsinki, Finland
L29	*Nostoc*	UK 222IIc	*Peltigera* sp. Mikkeli, Finland
L30	*Nostoc*	113.5	*Nephroma arcticum*, Finland
L31	*Nostoc*	UK 92Ic	*Peltigera* sp. Hitonhaudan rotko, Finland
L32	*Nostoc*	UK 89	*Peltigera* sp. Hitonhaudan rotko, Finland
L33	*Nostoc*	UK 81I	*Peltigera* sp. Scotland
L34	*Nostoc*	UK 222Ib	*Peltigera* sp. Mikkeli, Finland
L35	*Nostoc*	UK 60II	*Peltigera* sp. Scotland
L36	*Nostoc*	N135.9.1	lichen, unknown
L37	*Nostoc*	N138	lichen, unknown
L38	*Nostoc*	UK 104	*Peltigera* sp. Teeri-Lososuo, Kuhmo, Finland
L39	*Nostoc*	UK 220Ib	*Peltigera* sp. Mikkeli, Finland
L40	*Nostoc*	N134.1	lichen, unknown

**Figure 1:**
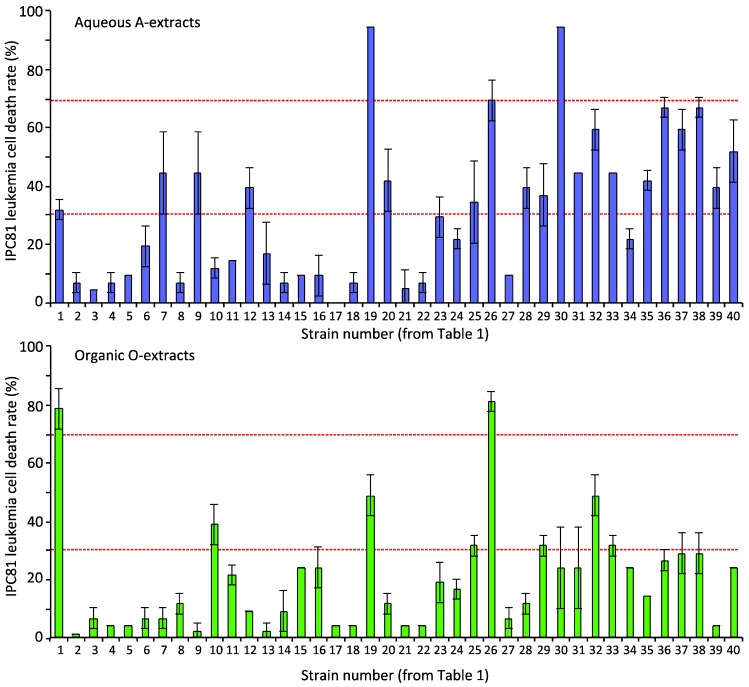
Leukemia cell death induced by cyanobacteria extracts. IPC-81 cells were incubated with extracts from a 5-mg biomass/mL cell suspension for 24 h before fixation in 2% buffered formaldehyde (pH 7.4). The X-axis gives the strain numbers (see Table 1 for details). Cell death was assessed by microscopic assessment of the surface and nuclear morphology. Horizontal lines show 30% (low) and 70% (high) apoptosis levels. A: aqueous; O: organic.

Twenty-eight extracts showed apparent apoptosis-inducing activity (a cell death rate above 30%); 20 were aqueous extracts, and eight were organic extracts (Figure 1). Four extracts (L19-A, L30-A, L1-O and L26-O) induced apoptosis of IPC-81 cells by over 70%. In several strains, both extracts induced apoptosis, such as L1, L19, L26 and L32. This indicated either two bioactive compounds or one compound present in both extracts. The present selection of cyanobacteria appeared to be a good resource for discovering anti-AML compounds.

**Figure 2:**
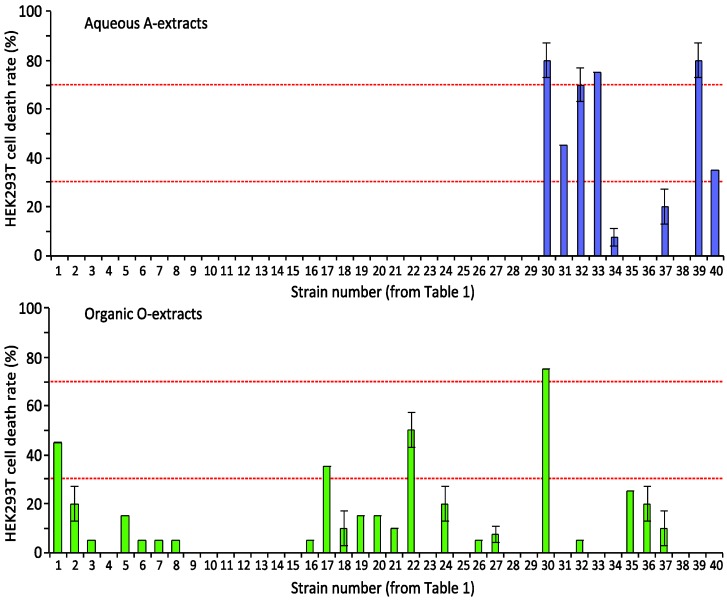
Human embryonic kidney (HEK293T) cell death induced by cyanobacteria extracts. HEK293T cells were incubated with extracts from a 5-mg biomass/ml cell suspension for 24 h before fixation in 2% buffered formaldehyde (pH 7.4). Cell death was assessed by microscopic assessment of surface and nuclear morphology. Horizontal lines show 30% (low) and 70% (high) apoptosis levels. A: aqueous; O: organic.

In order to reveal selectivity towards leukemia cells, we next tested the extracts for apoptosis induction in the human embryonic kidney cell line HEK293T (Figure 2), which can indicate whether a compound has non-specific toxicity. Six aqueous and four organic extracts exhibited toxicity (>30% cell death) to HEK293T. One strain, L30, showed very strong activity in both extracts. The extracts of L19-A-O, L26-A-O and L36-A that induced AML-cell death exhibited no toxicity to the HEK293T cells. This suggested that strains L19, L26 and L36 contain one or more compounds that preferentially induce cell death in AML-cells. Contrary to this, the organic extracts, L17-O and L22-O, revealed strong toxicity towards HEK293T cells, but not towards IPC-18 cells. Based on these two screenings (Figure 1 and Figure 2), we conclude that the cyanobacteria samples contained diverse bioactive compounds, some of which apparently are able to distinguish between AML cells and normal fibroblasts.

#### 2.1.2. The Detection of Known Bioactivities

Cyanobacteria produce large amounts of bioactive compounds able to induce cell death in mammalian cells, such as the liver toxins, microcystins and nodularins [26,27,28,29]. We have previously found high amounts of the metabolite adenosine in diatoms [30] and cyanobacteria [31], and adenosine can induce AML cell apoptosis [32]. It was necessary to establish the presence of these activities in the extracts with anti-AML activity. Whereas adenosine-mediated activity can be eliminated by enzymatic conversion of adenosine to inosine by adenosine deaminase, the microcystin-like activity can only be detected by LC-MS or cell assays.

**Figure 3:**
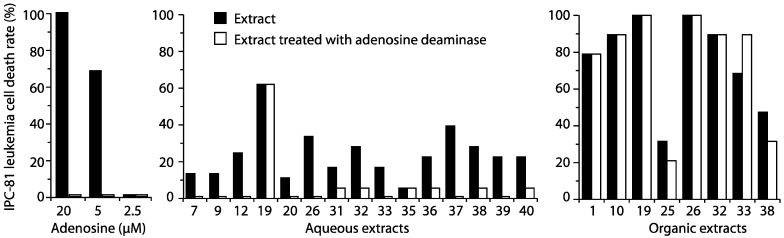
The presence of adenosine deaminase-sensitive compounds in cyanobacterial extracts. Cyanobacterial extracts were added to normal medium or medium containing adenosine deaminase and left to incubate at 30 min before the addition of IPC-81 cells. The cells were further incubated for 24 h before fixation and assessment of cell death, as described in the legend for Figure 1. As a control, adenosine was added to the medium with or without adenosine deaminase (**left panel**).

First, adenosine deaminase was used to remove adenosine from the AML death-inducing extracts. We found that some, but not all, extracts lost their apoptosis-inducing ability after this treatment (Figure 3) and that the adenosine-like activity mostly resided in the aqueous extracts. We concluded that the bioactive compounds in the adenosine deaminase-resistant extracts, like L19-A, and most of the organic apoptogenic extracts were unrelated to adenosine, but the activity could be due to adenosine analogs being resistant to adenosine deaminase.

**Figure 4:**
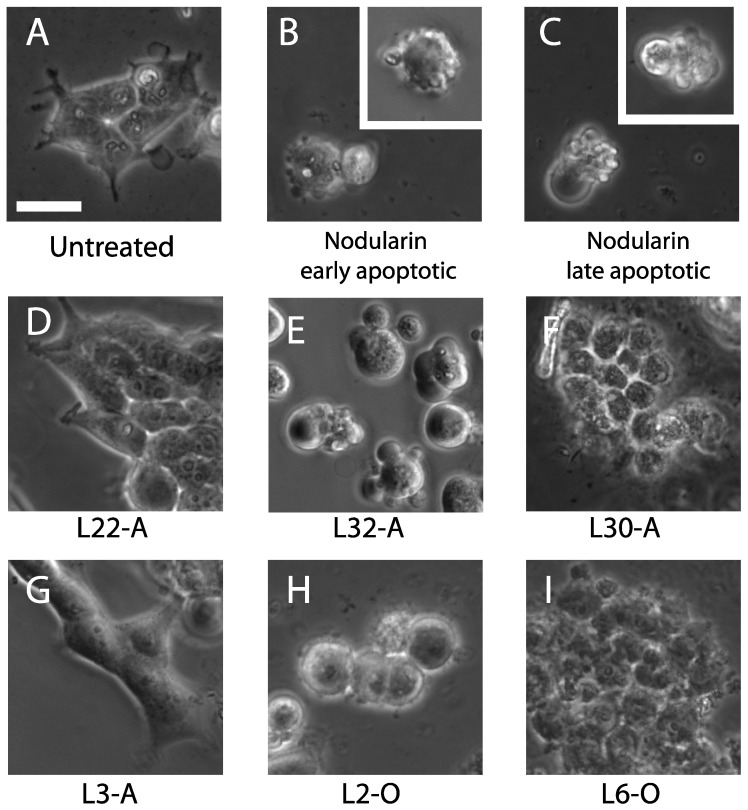
Cell death morphology in OATP1B3-transfected HEK293T cells treated with cyanobacterial extracts. HEK293T cells were transfected with OATP1B3 cells, as described in the Methods section, and treated with 5 mg/mL cyanobacterial extracts for 90 min before fixation in 2% buffered formaldehyde. (**A**,**D**,**G**) Normal morphology; (**D**,**G**) after treatment of extracts showing no bioactivity; (**B**,**C**) early and late apoptotic cells induced by 800 nM nodularin; (**E**) the nodularin-like response from cyanobacterial extract; (**H**) cell rounding and small blebs, which could be the signs of early apoptosis; (**F**,**I**) severe necrosis, perhaps induced by membrane-damaging compounds. A-extracts (**D**–**G**) are aqueous, and O-extracts (**H,I**) are organic. The scale bar represents 15 μm.

Although microcystins and nodularins are mainly dependent on transport into the cells to exert their pro-apoptotic activity, they can induce cell death in non-hepatocytes in very high concentrations. To ensure that we did not mistake microcystin-like activity for anti-AML activity, we took advantage of the need of specific transporters for such toxins to enter the cells [33,34]. Moreover, microcystin and nodularin induce the typical death morphology in OATP-transfected cells, with cell rounding and polarized budding [35]. We transfected HEK293T cells with the organic anion transporter protein, 1B3 (OATP1B3), which increased cell sensitivity towards microcystin and nodularin. The HEK293 cells were chosen over IPC-81 cells, since they are easier to transfect and display a distinct morphology upon treatment with microcystin and nodularin (see Figure 4B,C). We used the cell death morphology to identify microcystin-like activity (Figure 4). This revealed microcystin-like activity in six of the aqueous extracts and none of the organic extracts (Table 2). Microcystin-like apoptotic activity was found mostly in extracts from lichen-symbiotic cyanobacteria (Table 1 and Table 2). Some extracts, like L32-A, induced IPC-81 cell death, which could be eliminated by adenosine deaminase treatment (Figure 3), in addition to microcystin-like activity, evident in OATP1B3-transfected HEK293T cells (Figure 4E). The mild cytotoxicity seen in non-transfected HEK293T cells (Figure 2) could be a response to extremely high doses of microcystin or nodularin. There were also several cases where the cell death morphology was the same in OATP1B3 transfected and non-transfected HEK293T cells, as was the case for L30-A (Figure 4F).

To confirm the presence of microcystins/nodularins, cyanobacteria extracts were analyzed with LC-MS (Table 2). Although there was a good overall correlation, there were some cases where the LC-MS data failed to match the cell-based assay. The extracts of L7-A, L36-A, L38-A, L32-O, L33-O and L38-O, which were shown to contain microcystin by LC-MS, had no effect on triggering the apoptosis of HEK293T cells. This demonstrates the strength of both methods: LC-MS has higher sensitivity towards toxins, can detect very low amounts and will not be affected by the presence of toxin inhibitors [36]. However, the cell-based assay can detect activity from unknown toxin variants.

#### 2.1.3. Apoptosis-Inducing Features of Cyanobacteria Extracts

The seven most potent cyanobacteria extracts were tested to find the EC_50_ value in IPC-81 cells (Table 3). Both aqueous and organic extracts of L26 contained potent apoptosis-inducing activity and L26-O also exhibited the lowest EC_50_ among the seven extracts. In addition, L1-O and L30-A also showed low EC_50_ values against IPC-81 cells (Table 3). However, these extracts also induced HEK293T apoptosis at the initial screening, but this activity was not present in dilutions corresponding to 5–7 times the IC_50_ values for the IPC-81 cells (data not shown).

To get a better impression of the anti-AML activities in the extracts, we tested the most potent extracts for the ability to induce cell death in the human patient-derived AML cell lines, Molm-13. This cell line has internal tandem duplication of the tyrosine kinase, FLT3 [37,38], which is associated with poor outcome and could give an indication on whether the anti-AML activity identified in the IPC-81 screen (Figure 1) is also active towards more malignant AML variants. We found that extracts L30-A and L1-O were more potent towards these cells than the rat-derived IPC-81 cells. This emphasizes the potential of these cyanobacterial strains to provide useful compounds in the development of cancer therapy.

**Table 2:** The presence of microcystin and microcystin-like activity in selected cyanobacteria extracts.

Extract	Microcystin-like activity in OATP-transfected HEK293 cells	Microcystins by LC-MS (nM)
L1-A	−	−
L7-A	−	3.9
L9-A	−	−
L12-A	−	−
L19-A	−	−
L20-A	−	−
L26-A	−	−
L30 A	ND	3.8
L31-A	+	2100
L32-A	++	2700
L33-A	+	5100
L35-A	/	2600
L36-A	−	620
L37-A	+	13,000
L38-A	−	54
L39-A	+	2200
L40-A	+	1300
L1-O	−	−
L10-O	−	−
L19-O	−	−
L25-O	−	−
L26-O	−	−
L32-O	−	43
L33-O	−	89
L38-O	−	5.6

The + signifies the presence of bioactivity-inducing microcystin-like apoptotic morphology in OATP1B3-transfected HEK293 cells (second column) or the detection of microcystin or nodularin by LC-MS (third column). +, present, but less than 30% apoptosis; ++, strong (30%–100% apoptosis); −, absent; /, unknown; ND = not determined due to necrotic morphology.

**Table 3:** The involvement of the chemotherapy resistance gene in apoptosis caused by potent cyanobacteria extracts. A comparison of the EC_50_ values of potent cyanobacterial extracts against IPC-81 and Molm-13 cell lines.

Extract	EC_50_ (IPC-81)	Bcl-2 ^a^	p53 ^b^	EC_50_ (Molm-13)
L19-A	2.4	-	0	2.1
L26-A	>3.1	-	0	>3.1
L30-A	0.8	-	-	<0.5
L36-A	>4.8	-	0	>4.8
L1-O	0.3	-	-	<0.3
L26-O	<0.3	+	0	<<0.3
L30-O	ND	+	ND	ND

^a^ -, extract not able to overcome resistance from Bcl-2; +, extract induces cell death also in cells overexpressing Bcl-2; ^b^ 0, no change in cell response dependent on the p53 status; −, absence of p53 protects cells from cyanobacterial extract; +, absence of P53 sensitizes cells to cyanobacterial extract; ND, not determined.

We next checked whether the apoptogens of the potent cyanobacteria extracts could overcome some oncogenic features typical for both AML and other cancers. Bcl-2 is a key regulator of cell death, and the overexpression of Bcl-2 results in chemoresistance and increased tolerance to apoptogens [39]. We therefore tested our extracts on IPC-81 cells with enforced expression of Bcl-2 (Table 3) and found that these cells were resistant to all A extracts, but to a lesser degree to the O extracts from strain L26 and L30. This indicated that apoptosis-inducing activities in L26-O and L30-O partly overcome the chemotherapy resistance triggered by the overexpression of Bcl-2. It should be noted that L30-O also induced substantial HEK293T cell death (Figure 2).

p53 is well known as a tumor suppressor protein, which upon DNA damage, arrests cell growth and initiates apoptosis. Tumor suppression is severely reduced if the *TP*53 gene is mutated and the p53 protein is deficient or absent [40]. In this experiment, we exposed the human leukemia cell line, Molm-13, with functional p53 (wt) or constitutively expressing siRNA against p53 mRNA (shp53). This could reveal if the cyanobacterial apoptogens could overcome chemoresistance in p53-deficient cells. We found that the cell death induced by L30-A depended on p53. On the other hand, the apoptogen(s) in extract 1-O were more potent in cells with reduced p53 activity than in cells with intact p53 (Table 3). For the other extracts, there was little change in apoptosis-induction between the two cell types.

### 2.2. Discussion

A high number of the cyanobacterial extracts screened in this study contained apoptosis-inducing activity in acute myeloid leukemia cells, and some of them were also able to overcome typical features related to chemotherapy resistance. Our previous screenings also showed cyanobacteria as a prolific source of cytotoxic and anti-leukemic activities [41,42,43,44]. In addition, the continuous discovery of novel compounds from cyanobacteria [45,46,47,48,49] and the huge biosynthetic potential based on ribosomal and non-ribosomal biosynthetic gene clusters discovered in recent whole genome sequencing projects [50,51] make cyanobacteria an important group of micro-organism to be used as a source of new drug leads. With the vast number of different metabolites produced by cyanobacteria, often by related enzymes acting in concert [52], it is likely that cyanobacteria strains with a similar genetic background can produce different bioactive compounds. Small changes in the structure of such a compound can have a major impact on the bioactivity and mechanism of action on mammalian cells.

We detected apoptosis-inducing activity in more than fifty percent of the cyanobacteria strains (Figure 1 and Figure 2) and leukemia-selective bioactivity in 28 cyanobacteria extracts. Based on resistance to adenosine deaminase, we concluded that the bioactivity of most of the extracts was not due to adenosine or its analogs [32]. We could not exclude the possibility that some of our hits may be adenosine analogs. However, they retained their activity after adenosine deaminase treatment, suggesting that they will not be metabolized by adenosine deaminase in the blood.

Among the potent anti-leukemic cyanobacteria strains, three (L1, L19 and L26) were benthic marine cyanobacteria and two (L30 and L36) were from lichen. Benthic cyanobacteria have been previously shown to contain interesting bioactivities [41,42,43,44] in contrast to the planktonic, which often contain microcystins [14,15]. Lichen-associated cyanobacteria are known to produce the hepatotoxins, microcystins and nodularin [53], which can cause fatal injuries, such as liver damage [54], if they reach circulation. Despite this, we found anti-leukemic activity in lichen-associated strains (e.g., L32, L33, L36 and L38) (Table 1 and Table 2, Figure 1) and, thus, conclude that also the lichen-associated cyanobacteria are a new resource for finding anti-AML compounds and could be included in screens for pharmaceuticals.

We also found two extracts, L26-O and L30-O, from strains collected from a gastropod and lichen, respectively, which could partly overcome the chemoresistance mediated by enforced expression of Bcl-2 in the IPC-81 cells (Table 3). Bcl-2 is a well-known anti-apoptotic cell factor, which could result in chemotherapy resistance if overexpressed in the cancer cells [55,56]. Although the apoptosis-inducing activities in the remaining extracts were effectively blocked by Bcl-2 (Table 3), we cannot exclude the possibility that these extracts contain compounds that can become important leads in drug development.

Another oncogenic factor important in cancer chemotherapy is deleted or non-functional p53. In our study, the p53 mRNA in Molm-13 cells was silenced with the anti-sense RNA, and these cells are more resistant to apoptogens [57]. Interestingly, the extract, L1-O, induced more apoptosis in Molm-13 with silenced p53 cells than the wild-type, suggesting that L1-O could overcome the chemotherapy resistance caused by absent or non-functional p53, which is common in several cancers [58]. In addition, some extracts were more potent towards the Molm-13 cells than the IPC-81 cells (Table 3). The IPC-81 cells have a very rapid doubling rate of about 12 hours and are thus very sensitive towards drugs acting on the mitotic machinery. The extracts more potent towards Molm-13 cells could contain substances able to selectively attack the Achilles’ heel of malignant leukemia cells [59], leaving normal cells, like HEK293T cells, unaffected.

## 3. Experimental Section

### 3.1. Reagents

The main methanol and formaldehyde were from VWR (West Chester, NY, USA). Horse serum was supplied by EuroClone Life Sciences Division (Milan, Italy), and fetal bovine serum was from Invitrogen, (Carlsbad, CA, USA). Hoechst #33342 was from Polysciences, Inc. (Warrington, PA, USA). WST-1 was from Roche diagnostics (Basel, Switzerland). All other chemicals reagents, including cell culture media, were from Sigma (Sigma-Aldrich Co. LLC, St. Louis, MO, USA).

### 3.2. Isolation, Cultivation and Identification of Cyanobacteria

The cyanobacteria strains used in this study (see Table 1) were isolated from marine habitats (27 strains) and from symbiotic lichen (13 strains). The strains were identified based on their morphology according to Castenholz (2001) [60] and sequencing of the 16S rRNA gene, as previously detailed [61,62]. All cyanobacteria were grown in Z8X nitrogen free medium at 20 °C for 21 days under continuous light at a photon irradiance of 15 µmol·m^−2^·s^−1^. The cells were harvested by centrifugation at 10,000× *g* for 5 min.

### 3.3. Preparation of Cyanobacterial Extracts for Cell Experiments

Freeze-dried cyanobacteria biomass (20 mg) was suspended in 1.5 mL of methanol:water:dichloromethane (1:1:1). After homogenization, the suspensions were centrifuged for 10 min at 20,000× *g*. The upper and lower layers were collected separately and the remaining pellet re-extracted in the same manner. The extracts from the two extractions were combined, and the aqueous and organic layers evaporated in a vacuum centrifuge. The aqueous extracts were dissolved in 160 µL of 25% aqueous DMSO, and the organic extracts were dissolved in 40 µL of 100% DMSO and kept at −20 °C until bioassays.

### 3.4. Cell Lines and Maintenance

The rat promyeloid leukemia wild-type cell lines, IPC-81 [24] and IPC-81 with enforced expression of Bcl-2 [63], were cultured in Dulbecco’s modified Eagle’s medium (DMEM) supplemented with 10% (v/v) horse serum. The human acute monocytic leukemia cells, Molm-13 [37], were cultured in RPMI medium supplemented with antibiotics and 10% fetal bovine serum. Human embryonic kidney cells (HEK293T) were cultured in DMEM supplemented with 10% fetal bovine serum. When the cells reached 80% confluence, they were detached by mild trypsin treatment (0.33 mg·mL^−1^ trypsin for 5 min at 37 °C), washed and reseeded in fresh medium to 25% confluence. Enforced expression of OATP1B3 along with green fluorescent protein (GFP) by calcium phosphate transfection of 70% confluent HEK293T cells for 6 h. After 20 h, the cells were detached and seeded in 48-well tissue culture plates (30,000 cells/well), left to attach, and experiments were conducted. The vector for OATP1B3 [64] was a gift from Dietrich Keppler, German Cancer Research Center, Division of Tumor Biochemistry, Heidelberg, Germany. All cell media were supplemented with 50 U·mL^−1^ of penicillin and 0.05 mg·mL^−1^ of streptomycin.

### 3.5. Determination of Cell Death

For assays of cell death, the leukemia cells were centrifuged at 160× *g* for 4 min, suspended in fresh medium, and 1.5 × 10^4^ cells were seeded in 0.1 mL in a 96-well tissue culture plate. The HEK cells were detached by mild trypsin treatment, centrifuged, seeded in 48 wells (30,000 cells/well) and left overnight to attach before experiments. Extracts, toxins or solvents were added to the cells, and the plates were kept at 37 °C in a humidified atmosphere with 6% CO_2_ overnight before stopping the experiment by fixation of the cells in 2% buffered formaldehyde (pH 7.4) with 0.01 mg·mL^−1^ of the DNA-specific fluorescent dye, Hoechst 33342. Apoptosis was scored by differential interference contrast and fluorescence microscopy (Axiovert 35M, Carl Zeiss, Oberkochen, Germany), as previously described [43]. In some experiments, the WST-1 viability assay was used to score the metabolic capacity of treated cells. However, when using extracts containing pigments, we found the microscopic analyses more reliable.

### 3.6. Hydrolysis of Extracts with Adenosine Deaminase

To eliminate adenosine from the extracts, 1 µL of organic or 4 µL of aqueous extract was added to 50 U adenosine deaminase in a total volume of 50 µL DMEM medium. After 30 min, 50 µL of IPC-81 cells were added and experiments conducted as described above. Adenosine deaminase itself did not induce apoptosis. As a positive control, we added adenosine up to 20 µM with or without adenosine deaminase to the cells. The adenosine deaminase completely removed the apoptotic potential of adenosine (Figure 3).

### 3.7. Analysis of Microcystins with LC-MS

Between 7 and 31 mg of freeze-dried cells of forty cyanobacteria strains were extracted with 1.5 mL extracting solvent, including methanol, water and dichloromethane, at a rate of 1:1:1. In addition, 200 µL of 0.5 mm glass beads (Scientific Industries, New York, NY, USA) were added in the 2-mL tubes for homogenization. This was carried out on a Fast Prep homogenizer (FP120, Bio 101, Savant) for 60 s at a speed value of 6 m·s^−1^. Extracts were centrifuged for 5 min at 20,000× g. Then, the upper aqueous and lower organic layer were collected and evaporated. After evaporation, the aqueous layer was resuspended in 25% aqueous acetonitrile, and the organic layer was dissolved in 100% acetonitrile before LC-MS analyses. LC-MS analyses were performed with an Agilent 1100 Series LC/MSD Ion Trap XCT Plus System (Agilent Technologies, Palo Alto, CA, USA) using a Phenomenex Kinetex C18 (150 × 2.1 mm, 5 µm, Phenomenex, Torrance, CA, USA) LC-column. For the quantification of microcystins, the extraction was made as described above, except that the organic extract was dissolved in 100 µL of DMSO and the aqueous extract in 160 µL of 25% DMSO. Microcystin-LR and Microcystin-RR were used as the standard. Analysis was done as previously described [65].

## 4. Conclusions

We conclude that both marine and lichen-associated cyanobacteria can provide substances that eventually could become lead compounds for cancer therapy. Among the active cyanobacteria extracts, L26-O presented the highest apoptosis-inducing activity, could partly overcome the chemotherapy resistance caused by the Bcl-2 protein and was highly potent also against the human leukemia patient-derived cell line, Molm13. If the apoptogens in the anti-AML positive extracts could be isolated and identified, there could be several potent leads for developing improved cancer therapy.
